# Charge-transfer interaction of aspartame and neotame with several π-acceptors: Stoichiometric data

**DOI:** 10.1016/j.dib.2021.107092

**Published:** 2021-04-24

**Authors:** Abdel Majid A. Adam, Tariq A. Altalhi, Hosam A. Saad, Amnah M. Alsuhaibani, Moamen S. Refat, Mohamed S. Hegab

**Affiliations:** a Department of Chemistry, College of Science, Taif University, P.O. Box 11099, Taif 21944, Saudi Arabia; b Department of Physical Sport Science, Princess Nourah bint Abdulrahman University, Riyadh, Saudi Arabia; c Deanship of Supportive Studies (D.S.S.), Taif University, P.O. Box 11099, Taif 21944, Saudi Arabia

**Keywords:** Charge-transfer interaction, Aspartame, Neotame, Stoichiometry, Job's continuous variation method

## Abstract

This data article is related to a research paper entitled ``Correlations between spectroscopic data for charge-transfer complexes of two artificial sweeteners, aspartame and neotame, generated with several π-acceptors'' [J. Mol. Liq. 333 (2021) 115904] [1]. Herein we present stoichiometric data of charge-transfer (CT) complexes generated from the interaction between aspartame and neotame with three π-acceptors in methanol solvent at room temperature. The investigated π-acceptors were picric acid (PA), chloranilic acid (CA), and 2,3-dichloro-5,6-dicyano-p-benzoquinone (DDQ), where the methods used to determine the stoichiometry of the CT interaction were the spectrophotometric titration method and the Job's continuous variation method.

**Specifications Table**

**Table:** 

Subject	Chemistry
Specific subject area	Charge-transfer (CT) complexation
Type of data	Graph/Plot
How data were acquired	OriginPro 9 software, UV/Vis spectrophotometer
Data format	Raw and analysed
Parameters for data collection	All data were collected on CT complexes generated in methanol solvent at room temperature using analytical grade chemicals.
Description of data collection	Solutions of aspartame and neotame, and each acceptor dissolved in methanol solvent were mixed and the resultant CT complexes were scanned using a UV/Vis spectrophotometer. These UV/Vis spectra were compared with those from the free donors and acceptors alone to verify the stoichiometry of the interaction.
Data source location	Department of Chemistry, College of Science, Taif University, Taif, Saudi Arabia
Data accessibility	Data are available with the article.
Related research article	A.M.A. Adam, T.A. Altalhi, H.A. Saad, A.M. Alsuhaibani, M.S. Refat, and M.S. Hegab, Correlations between spectroscopic data for charge-transfer complexes of two artificial sweeteners, aspartame and neotame, generated with several π-acceptors, J. Mol. Liq. 333 (2021) 115904. https://doi.org/10.1016/j.molliq.2021.115904

## Value of the Data

• Investigating the charge-transfer (CT) properties of the artificial sweeteners, aspartame and neotame may be useful toward improving their uses and applications.
• Verifying the stoichiometry of the interaction between the artificial sweeteners, aspartame and neotame with different acceptors is very important to understand the mode of the interaction of these sweeteners toward improving their uses and applications.
• The most important and useful methods to determine the stoichiometry of the interaction between molecules are Job's continuous variation method and the spectrophotometric titration method. These methods are easily performed.

## 1 Data Description

**Fig. 1:**
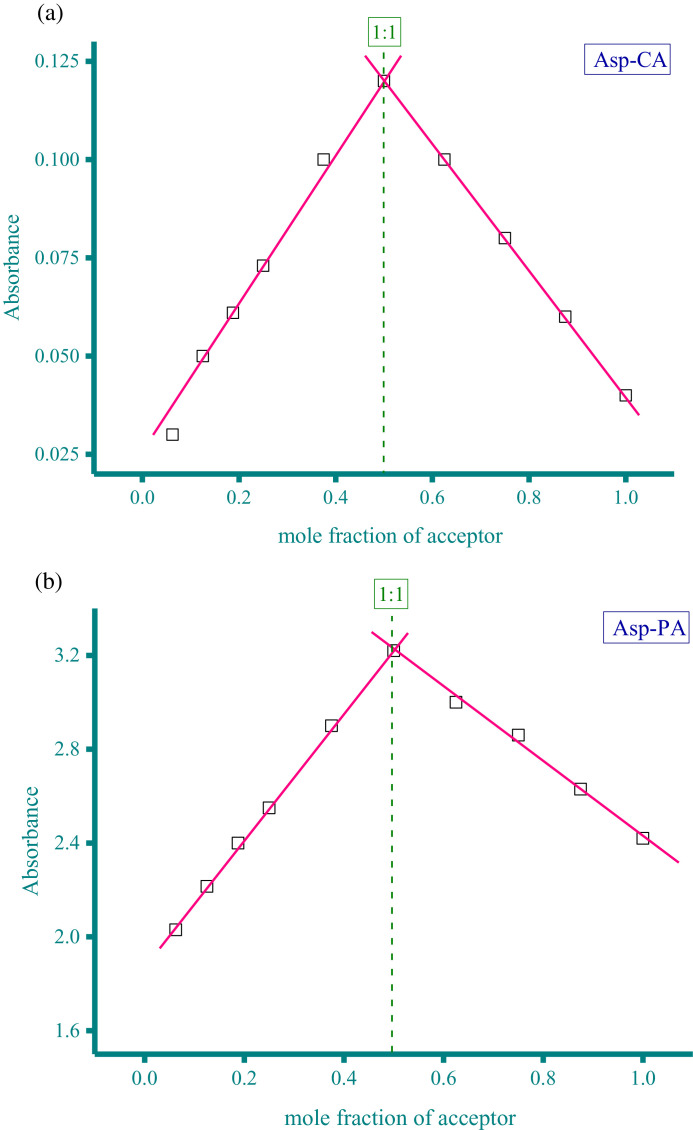

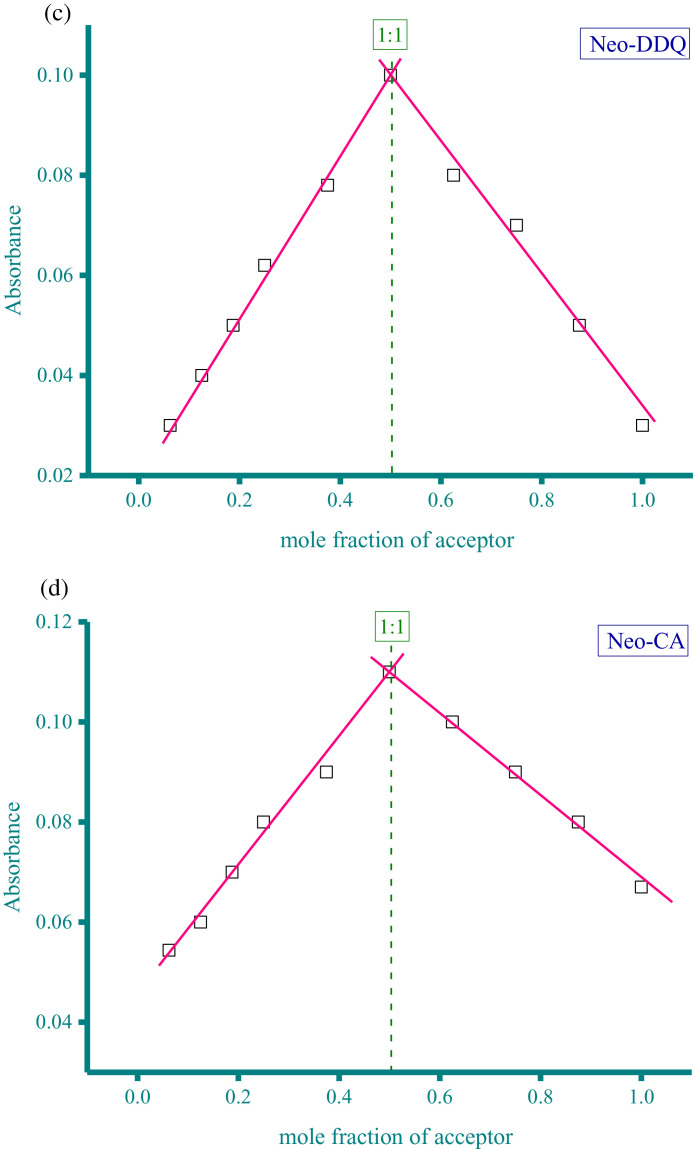

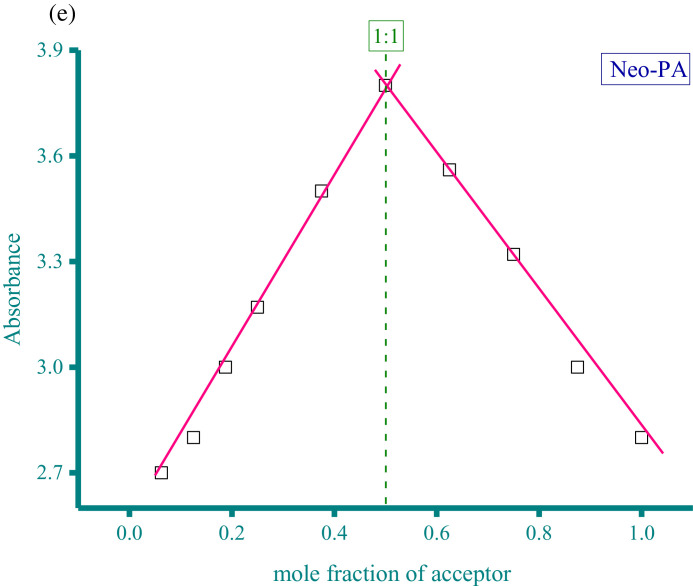
a. Composition between Asp and CA determined by Job's continuous variation method. b. Composition between Asp and PA determined by Job's continuous variation method. c. Composition between Neo and DDQ determined by Job's continuous variation method. d. Composition between Neo and CA determined by Job's continuous variation method. e. Composition between Neo and PA determined by Job's continuous variation method.

**Fig. 2:**
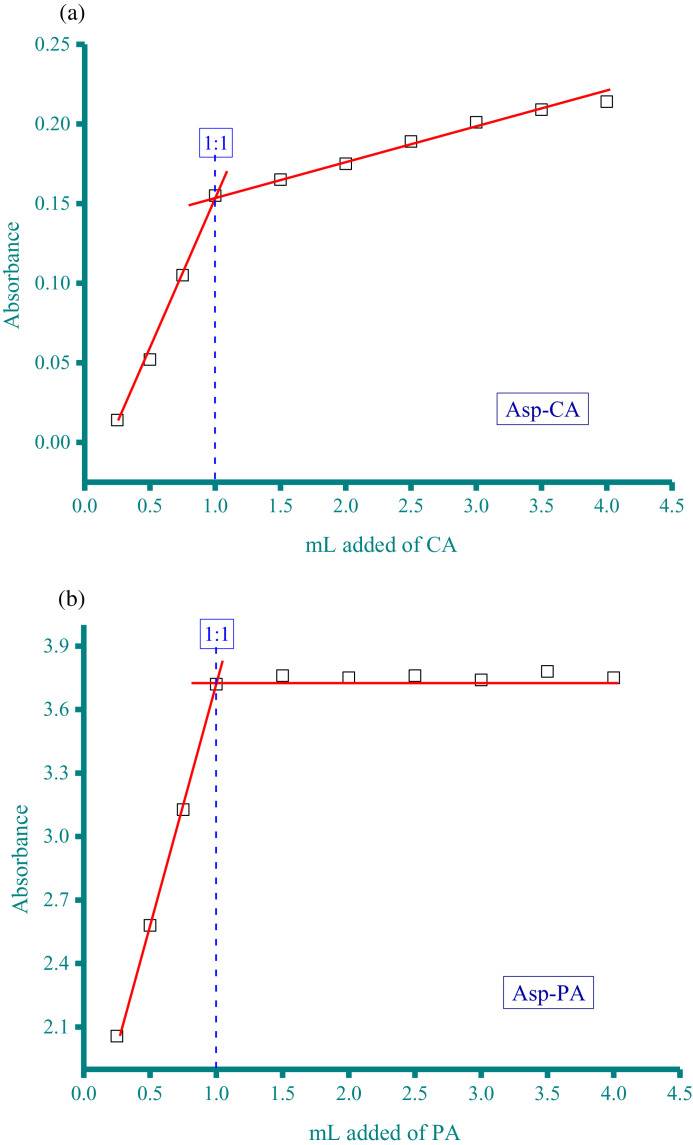

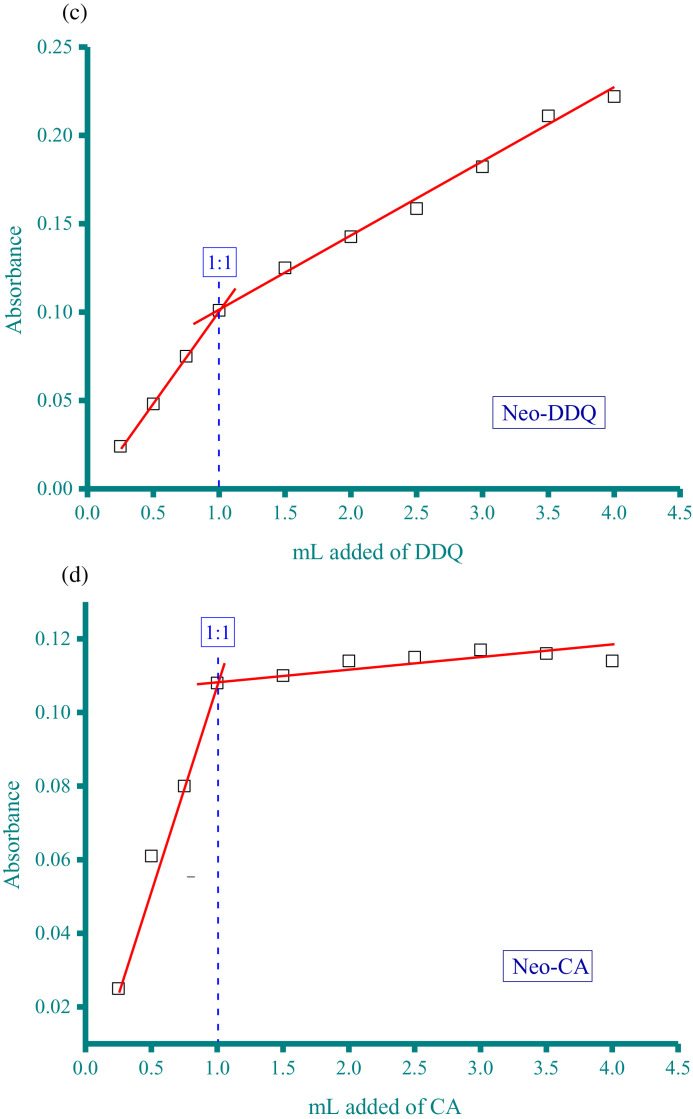

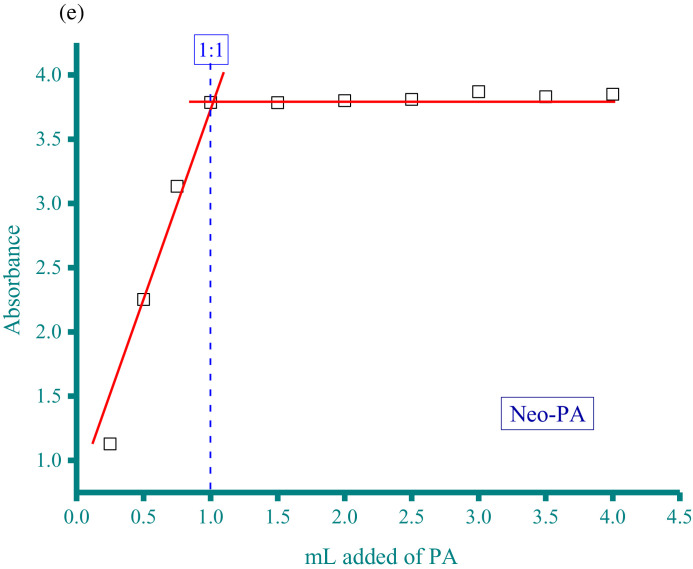
a. Composition between Asp and CA determined by spectrophotometric titration method. b. Composition between Asp and PA determined by spectrophotometric titration method. c. Composition between Neo and DDQ determined by spectrophotometric titration method. d. Composition between Neo and CA determined by spectrophotometric titration method. e. Composition between Neo and PA determined by spectrophotometric titration method.

Methanolic solutions of aspartame (Asp) and neotame (Neo) as donors at concentration of 5 × 10^−4^ M were each individually mixed with three acceptor solutions in methanol solvent at the same concentration (5 × 10^−4^ M). These acceptors were CA, PA, and DDQ. The resultant donor-acceptor systems were Asp−CA, Asp−PA, Neo−DDQ, Neo−CA, and Neo−PA, which were scanned using a UV/Vis spectrophotometer (The UV/Vis spectra dataset were provided as separated Excel sheet). The stoichiometry of the interaction between the donors (Asp and Neo) and the acceptors (CA, PA, and DDQ) was obtained using i) Job's continuous variation method [2], and ii) the spectrophotometric titration method [3]. Fig. 1(a-e), respectively, contain the curves generated for the five systems using Job's continuous variation method (the raw data associated with this figure were based on one measurement, and listed in Table 1). Fig. 2(a-e), respectively, contain the curves generated for the five systems using spectrophotometric titration method (the raw data associated with this figure were based on one measurement, and listed in Table 2).

**Table 1:** Absorbance of the Asp−CA, Asp−PA, Neo−DDQ, Neo−CA, and Neo−PA systems at different mole fraction of acceptor (the raw data for Fig. 1).

	Absorbance				

Mole fraction of acceptor	Asp−CA	Asp−PA	Neo−DDQ	Neo−CA	Neo−PA
0.0625	0.03	2.03	0.03	0.0544	2.7
0.125	0.05	2.215	0.04	0.06	2.8
0.1875	0.061	2.4	0.05	0.07	3
0.25	0.073	2.55	0.062	0.08	3.17
0.375	0.1	2.9	0.078	0.09	3.5
0.5	0.12	3.22	0.1	0.11	3.8
0.625	0.1	3	0.08	0.1	3.56
0.75	0.08	2.86	0.07	0.09	3.32
0.875	0.06	2.63	0.05	0.08	3
1	0.04	2.42	0.03	0.067	2.8

**Table 2:** Absorbance of the Asp−CA, Asp−PA, Neo−DDQ, Neo−CA, and Neo−PA systems at different acceptor volume (the raw data for Fig. 2).

	Absorbance				

mL added of acceptor	Asp−CA	Asp−PA	Neo−DDQ	Neo−CA	Neo−PA
0.25	0.014	2.057	0.024	0.025	1.128
0.5	0.052	2.58	0.048	0.061	2.252
0.75	0.105	3.127	0.075	0.08	3.133
1	0.155	3.72	0.101	0.108	3.786
1.5	0.165	3.76	0.125	0.11	3.784
2	0.175	3.75	0.1426	0.114	3.8
2.5	0.189	3.76	0.1586	0.115	3.81
3	0.201	3.74	0.1822	0.117	3.87
3.5	0.209	3.78	0.211	0.116	3.83
4	0.214	3.75	0.222	0.114	3.85

## 2 Experimental Design, Materials and Methods

### 2.1 Materials

Analytical-grade Asp (C_14_H_18_N_2_O_5_; 294.30 g/mol; purity ≥ 98%), Neo (C_20_H_30_N_2_O_5_; 378.47 g/mol; purity ≥ 98%) were bought from SUPELCO Analytical Company. PA (C_6_H_3_N_3_O_7_; 229.10 g/mol; purity ≥ 98%), CA (C_6_H_2_Cl_2_O_4_; 208.98 g/mol; purity ≥ 98%), and DDQ (C_8_Cl_2_N_2_O_2_; 227.00 g/mol; purity 98%) were bought from Sigma-Aldrich Chemical Company.

### 2.2 Methods

1. Donors (Asp, Neo), and acceptors (PA, CA, and DDQ) solutions were individually prepared at 5 × 10^−4^ M in methanol in 25-mL volumetric flasks.

2. The solubilized donor (Asp or Neo) (1 mL) was combined with each solubilized acceptor (PA, CA, or DDQ) (1 mL) and methanol (3 mL) in 5-mL glass tubes to generate the Asp−CA, Asp−PA, Neo−DDQ, Neo−CA, and Neo−PA systems.

3. The UV-visible spectra of the free compounds and prepared systems were collected at room temperature from 200 to 800 nm using a Perkin−Elmer Lambda 25 UV/Vis spectrophotometer and used to obtain the CT band (λ_CT_).

4. To verify the stoichiometry of the interaction between donors and each of the acceptors using the spectrophotometric titration method, the absorbances (λ_CT_) of 10 standard solutions with varied donor to acceptor molar ratios (from 4:1 to 1:4) were plotted against the volume of the acceptor in each standard solution.

**Table:** 

No. of the standard solution	1	2	3	4	5	6	7	8	9	10
Molar ratio (donor: acceptor)	4:1	2:1	1.3:1	1:1	1:1.5	1:2	1:2.5	1:3	1:3.5	1:4
Volume of the acceptor (mL)	0.25	0.5	0.75	1.0	1.5	2.0	2.5	3.0	3.5	4.0
Volume of the donor (mL)	1.0	1.0	1.0	1.0	1.0	1.0	1.0	1.0	1.0	1.0

5. To verify the stoichiometry of the interaction between donors and each of the acceptors using Job's continuous variation method, the absorbances (λ_CT_) of 10 standard solutions with varied molar fractions of donor and acceptor (C_donor_ + C_acceptor_) were plotted against the molar fraction of the acceptor in each standard solution.

**Table:** 

No. of the standard solution	1	2	3	4	5	6	7	8	9	10
Molar fraction of acceptor	0.0625	0.125	0.1875	0.25	0.375	0.5	0.625	0.75	0.875	1.0
Volume of the acceptor (mL)	0.25	0.5	0.75	1.0	1.5	2.0	2.5	3.0	3.5	4.0
Volume of the donor (mL)	3.75	3.5	3.25	3.0	2.5	2.0	1.5	1.0	0.5	0.0

## CRediT Author Statement

**Abdel Majid A. Adam:** Data curation, Writing – original draft, Writing – review and editing; **Tariq A. Altalhi:** Conceptualization, Methodology, Software, Supervision; **Hosam A. Saad:** Visualization, Investigation, Software, Validation; **Amnah M. Alsuhaibani:** Visualization, Investigation, Software, Validation; **Moamen S. Refat:** Conceptualization, Methodology, Software, Supervision; **Mohamed S. Hegab:** Visualization, Investigation, Software, Validation.

## Declaration of Competing Interest

The authors declare that they have no known competing financial interests or personal relationships that could be perceived to have influenced the work reported in this article.

## Appendix Supplementary materials

Image, application 1
